# Fine-Tuned Siamese Network with Modified Enhanced Super-Resolution GAN Plus Based on Low-Quality Chest X-ray Images for COVID-19 Identification

**DOI:** 10.3390/diagnostics12030717

**Published:** 2022-03-15

**Authors:** Grace Ugochi Nneji, Jingye Cai, Happy Nkanta Monday, Md Altab Hossin, Saifun Nahar, Goodness Temofe Mgbejime, Jianhua Deng

**Affiliations:** 1 School of Information and Software Engineering, University of Electronic Science and Technology of China, Chengdu 611731, China; ugochinneji@std.uestc.edu.cn (G.U.N.); jianhua.deng@uestc.edu.cn (J.D.); 2 School of Computer Science and Engineering, University of Electronic Science and Technology of China, Chengdu 611731, China; mh.nkanta@std.uestc.edu.cn (H.N.M.); temofeerics@gmail.com (G.T.M.); 3 School of Management and Economics, University of Electronic Science and Technology of China, Chengdu 611731, China; altabbd@uestc.edu.cn; 4 Department of Information System and Technology, University of Missouri St. Louis, St. Louis, MO 63121, USA; snnnm@umsl.edu

**Keywords:** adversarial learning, chest X-ray images, contrastive loss, COVID-19, deep learning, Siamese network, super-resolution

## Abstract

Coronavirus disease has rapidly spread globally since early January of 2020. With millions of deaths, it is essential for an automated system to be utilized to aid in the clinical diagnosis and reduce time consumption for image analysis. This article presents a generative adversarial network (GAN)-based deep learning application for precisely regaining high-resolution (HR) CXR images from low-resolution (LR) CXR correspondents for COVID-19 identification. Respectively, using the building blocks of GAN, we introduce a modified enhanced super-resolution generative adversarial network plus (MESRGAN+) to implement a connected nonlinear mapping collected from noise-contaminated low-resolution input images to produce deblurred and denoised HR images. As opposed to the latest trends of network complexity and computational costs, we incorporate an enhanced VGG19 fine-tuned twin network with the wavelet pooling strategy in order to extract distinct features for COVID-19 identification. We demonstrate our proposed model on a publicly available dataset of 11,920 samples of chest X-ray images, with 2980 cases of COVID-19 CXR, healthy, viral and bacterial cases. Our proposed model performs efficiently both on the binary and four-class classification. The proposed method achieves accuracy of 98.8%, precision of 98.6%, sensitivity of 97.5%, specificity of 98.9%, an F1 score of 97.8% and ROC AUC of 98.8% for the multi-class task, while, for the binary class, the model achieves accuracy of 99.7%, precision of 98.9%, sensitivity of 98.7%, specificity of 99.3%, an F1 score of 98.2% and ROC AUC of 99.7%. Our method obtains state-of-the-art (SOTA) performance, according to the experimental results, which is helpful for COVID-19 screening. This new conceptual framework is proposed to play an influential role in addressing the issues facing COVID-19 examination and other diseases.

## 1. Introduction

Coronavirus 2019 (COVID-19) is a unique type of coronavirus disease that is a pulmonary ailment instigated by severe pneumonia. It was first discovered in late 2019, becoming a universal epidemic and reaching over 418 million confirmed cases and 5.8 million deaths across the globe as of 17 February 2022 [1,2]. In the earliest discovery of this disease, the only efficient means of containing COVID-19 were washing and sanitizing the hands, wearing face shields, maintaining reasonable social distancing, testing the population and isolating the affected persons, as stated by the World Health Organization (WHO). Researchers and medical practitioners are currently developing vaccines as a cure for COVID-19. However, there is no vaccine with 100% availability to treat COVID-19 or medication to avert further spread. In alleviating the increasingly contagious disease, the need for early identification of COVID-19 is critical.

Now, reverse-transcription polymerase chain reaction (RT-PCR) is an acceptable procedure for diagnosing COVID-19; this test is performed manually to carry out viral nucleic acid examination by using nasopharyngeal and throat swabs [3]. RT-PCR is complex, time-consuming and contains sampling errors. Moreover, it requires multiple tests for a definitive result and has relatively low sensitivity. Defective equipment, the small number of clinical professionals and the swift accumulation of affected patients demand robotic screening applications. This type of application serves as an alternative for medical professionals to rapidly recognize individuals who have contracted the disease, thereby isolating them. The following alternative screening methods [4,5], which have been established for the COVID-19 process, are radiographs that employ either a chest X-ray radiograph or chest tomography (CXR or CT) to search for viral presence in the lungs.

Chest X-ray (CXR) [6,7] is an essential medical tool that plays a vital role in the earliest exploration of lung abnormalities. It can be used as an alternative solution for screening in detecting COVID-19 or validating other related diagnoses, and extremely skilled radiologists characterize the CXR images [8,9], searching for infectious lesions contributing to COVID-19. These typical features involve varieties of opacities and multi-focal in patients. However, manual comprehension of the delicate CXR digital image attributes is challenging, thereby demanding a professional in the field. Moreover, the rapid increase in individuals who have contracted this disease makes it challenging for the radiologist [10,11] to accomplish the diagnosis rapidly, thus creating a higher death rate [12,13]. A combination of CXR digital images gathered from various outpatient clinics, articles and previous research studies was used [14,15,16]. After the retroactive analysis of the earlier literature works, we noticed that previous research works had been accomplished with a small amount of CXR or CT images as input, causing the deep learning (DL) models to under-fit. However, with the high predominance of pneumonia, in comparison to the massive amount of healthy images, there are a small number of COVID-19 images accessible on which to create a deep learning model. Consequently, regarding CXR, DL methodologies for COVID-19 classification have been thoroughly investigated [17,18]. A public-domain deep convolutional neural network framework was proposed in [11], tailored to identify COVID-19 from chest radiography. Several methods [19,20,21,22] have been incorporated to resolve the COVID-19 diagnosis problem.

Meanwhile, these methods have some flaws in common:

1. Rather than recovering the displaced specific appearance information, they entail high computing costs, as well as requiring a large number of correctly annotated CXR images for model training, thereby restraining medical approval.

2. They focus exclusively on deeper neural networks [23,24,25,26], which leads to model complexity and high computational costs, rather than using the ability of a simple convolutional neural network to discover and maximize the distinctive characteristics, especially regarding the resource constraints of an AI-based COVID-19 diagnosis system.

However, in AI-based COVID-19 diagnosis, low resolution (LR) remains a significant challenge for large-scale CXR imaging applications. Naturally, super resolution (SR) provides an effective algorithm to reduce the resolution disparity dilemma because of its capability to generate high-frequency information [27]. In several studies, a GAN-based architecture can resolve the unavailability of images, which causes over-fitting in the CNN architecture. This study addresses the low-resolution AI-based COVID-19 diagnosis issue by generating super-resolution images with the generative adversarial network method and applying a COVID-19 identification algorithm in an incorporated framework termed the enhanced Siamese fine-tuned model with wavelet pooling strategy and modified enhanced super-resolution GAN plus based on low-quality images for COVID-19 identification (COVID-SRSNet). We carry out preprocessing to handle resolution mismatches by reshaping them to a targeted steady resolution of 224 × 224 pixels. This research aims to investigate low-resolution COVID-19 identification; for this, we considered all images that were low-resolution. Generally, after down-sampling, it is difficult to extract low-level characteristics; however, we used a GAN-based enhanced super resolution to reproduce HR correspondents from LR images to improve the network’s feature extraction capability. This method extracts the specific visual appearance details effectively. Our proposed algorithm adopts the super-resolution approach, thereby reducing the complexity of the problem and the time it takes to solve it. Additionally, to maintain a lightweight and far less complex architecture, a fine-tuned VGG19 twin network with wavelet pooling is applied to boost the feature extraction. The proposed architecture algorithm supplies good interpretability and sensitivity in comparison to existing methods.

The following points summarize the main significance of our article:

1. The enhanced Siamese fine-tuned model with wavelet pooling strategy and modified enhanced super-resolution GAN plus based on low-quality images for COVID-19 identification (COVID-SRSNet) sufficiently addresses the issues of resolution disparity by taking inconsistent input dimensions and reshaping them to a fixed resolution dimension using an image scale-based adaptive module.

2. COVID-SRSNet is utilized for effective image recapturing for the purpose of handling the problem of low-quality images and eliminating noisy presumable distortion caused by GAN as a result of the JPEG decompressing nature of the image format. It employs a residual-integrated-residual dense block to create real and genuine images. The relativistic network consists of a discriminator used to boost the identification details efficiently. The batch normalization layer is removed for regular training and performance, as well as to eliminate the noisy artifacts [28]. The perceptual loss is enhanced by extricating the distinct details before introducing the activation function to prevent detail scarcity [28]. In supporting the thorough network training, a minor initialization technique and residual scaling were adopted.

3. With the use of our modified Siamese network of fine-tuned VGG16 by incorporating a contrastive loss function and Euclidean distance, we efficiently classify our datasets into a binary class and multiple classes.

The remaining parts of the paper will survey related works in Section 2, whereas Section 3 will give a detailed explanation of the methodology. Descriptive information about the dataset, the implementation technicalities and experimental outcomes will be presented in Section 4. Section 5 will shed more light on the result validation and other related discussions. Section 6 presents the conclusions of this study.

## 2. Related Works

An in-depth analysis of the literature relevant to our research is introduced in this part. Firstly, we survey published works on the imaging-based screening of COVID-19 utilizing CXR or CT images. Secondly, we analyze various topics in conjunction with our novel framework, including generative adversarial network (GAN), Siamese convolutional neural network, identification and deep metric learning.

### Imaging-Based Diagnosis of COVID-19

This section provides a comprehensive review of published research on COVID-19 investigation using CXR or CT images. Deep neural networks are adopted to accurately diagnose a diversity of infectious and non-infectious diseases from medical imaging data, which recurrently outweighs human efforts [12]. To recognize images as COVID-19, healthy or pneumonia, a discriminative cost-sensitive learning (DCSL) was proposed in [13]. The model was trained on two datasets from the public domain [13,15]. The model had a sensitivity of 97.1%, accuracy of 97%, precision of 97% and F1 score of 91%. This degree of efficiency is achieved by training on labeled data and fine-tuning the system’s millions of parameters. Deep learning systems are being used in a variety of published research for COVID-19 diagnosis and screening. The ImageNet weights were pre-trained on a designed 18-layer residual network against 100 COVID-19 and 1431 pneumonia X-ray datasets [29]. Generally, COVID-19, healthy and viral pneumonia CXR images are the most commonly curated datasets [30]. The subject of the effectiveness of AI-based COVID-19 diagnosis is reported in [31], in which the authors focused on the role of the early detection of COVID-19 patients as a vital tool to mitigate the spread of the virus as well as ease the burden on clinicians. The authors reported their findings that deep learning models are a promising solution due their high sensitivity results compared to expert diagnosis. Lu et al. [32], who adopted a neural network approach for the prediction of intensive care unit admissions, concluded that biomarkers such as creatinine, C-reactive protein, etc., indicated momentary variations among admitted COVID-19 patients in the ward and transferred to the intensive care unit, in contrast to the patients not transferred.

A machine learning method is suggested in [33] to rule out routine blood tests as the sole data for COVID-19 diagnosis in the emergency unit, especially among adults. The authors of this study fused multicenter health data acquired from an emergency unit’s laboratory and their method achieved 97% specificity and 98% sensitivity. Aslan et al. [34] suggested a scheme of classifying COVID-19 chest computed tomography images using famous feature extraction CNN architectures such as AlexNet, ResNet18, ResNet50, Inceptionv3, Densenet201, Inceptionresnetv2, MobileNetv2 and GoogleNet and achieved the highest accuracy of 96.29%. The two key significances of their work involve the identification of machine learning hyper-parameters by using Bayesian optimization and ANN-based image segmentation.

Interestingly, the authors in [35] compared and quantified the preferences of selected patients for AI-based clinicians and human clinicians. A method of propensity similarity score matching was adopted to match similar demographic characteristics among two separate groups of respondents. The final report presented in this study showed that 95% of the respondents believed that the AI-based diagnosis technique achieves better accuracy with lower expenses compared to human-based clinician diagnosis.

Aslan et al. [36] suggested two deep learning frameworks for automatically detecting positive COVID-19 instances using chest CT images, which include lung segmentation as a preprocessing phase for the CT images, which are then passed as input to the proposed artificial neural network (ANN) architectures for automatic detection, with a hybrid model achieving 98.70% accuracy.

When a random forest classifier was adopted with important multimodal characteristics such as age, hypertension, gender, diabetes and cardiovascular disease, the authors reported 96% prediction accuracy. Different classification methods of prioritizing symptomatic patients for COVID-19 early detection using metadata such as age, gender and fever were proposed in [37]. This method achieved an average of 90% accuracy. Li et al. [38] formulated a DL model and a risk rating algorithm for the outcome of intensive care unit admission and death in the hospital. The ROC-AUC was utilized as a metric to evaluate the model’s performance. The authors discovered that these biomarkers were the leading ICU indicators, besides age, cardiac troponin and oxygen saturation, which were the main death indicators. A Bayesian CNN with weight reduction was advocated, utilizing ResNet-50 V2, as proposed in [16], where normal, bacteria, viral pneumonia and COVID-19 were the four classes included in the dataset. Furthermore, two of the COVID-19 instances were incorrectly classified using CNN and BCNN.

To lessen the black-box traits of deep learning, various saliency maps were utilized; conversely, the maps appeared to focus on some inexact areas not described in the article. Nevertheless, the study noted that calculating the prediction’s ambiguity could improve the model’s technique. A total of 50 images each for healthy and COVID-19 patients were obtained in [18] and utilized in three pre-trained [19,20,21] DL algorithms to detect COVID-19, obtaining better performance. A residual CNN with 18 layers and a sigmoid activation function pre-trained on ImageNet for classification was proposed in [22,23]. The authors reported that the algorithm obtained 72% sensitivity using 362 CXR images and 98% specificity. Nevertheless, while these models seem to produce great performance, model sensitivity is a primary concern due to the danger of misleading COVID-19 diagnosis results.

Several models are refined utilizing CT to examine COVID-19, as suggested in [8,9]. A UNet++ [24] approach to detect and examine COVID-19 lesions was proposed in [25]. Training the model on professionally annotated CT slices to obtain COVID-19 sections showed comparable achievement to professional radiologists, with 100% sensitivity. Using 300 images of COVID-19 patients, a dice scoring rate of 91.6% [26] was achieved to segment and analyze COVID-19. COVID-19 was segmented and quantified using a combination of commercial software and DL techniques in [27], with 99.6% AUC. A weight-shared twin ResNet-50 network was used for individual CT image slices, after which they were combined by max-pooling to produce a single feature vector used for the classification task in [28]. This algorithm utilized 285 images of healthy patients and 68 COVID-19 instances of confirmed infected cases to achieve an AUC of 96.0% [28]. Using 53 patients, these authors [39] tested the patch-based technique with an SVM classifier and obtained 100% specificity and 93% sensitivity. Five-fold cross-validation based on an infection size conscious random forest classifier approach was proposed in [40] using segmented scans to identify infection and lung areas, and then images were categorized based on infection size, which obtained an average of 94% AUC with 1027 healthy and 1658 COVID-19 images.

The first attempt to use a 3D neural network to segment lesions with a 2D ResNet network before classifying them as healthy or COVID-19 instances was proposed in [41]. This approach obtained 99% AUC on 128 healthy images and 154 COVID-19 images using two hospitals’ data. The use of a segmentation network and a ResNet-152 network model to classify CT slices was proposed in [42,43]. GradCAMs were then developed in [44] to illustrate the diseased region. On 1072 healthy and 183 COVID-19 images, this network model was trained using private and public data, attaining 98% AUC. The approach to segmenting infection areas using a deep learning algorithm was suggested in [45]. Patches of infected areas were considered the input data to the ResNet-18 network proposed in [45], taking into account the distances from the lung’s edge as images are classified into healthy and COVID-19 influenza images. The network model attained an average of 86.7% accuracy on 30 COVID-19 and 60 non-COVID-19 images.

Another interesting work was proposed in [46], where the inception model was utilized using an in-house dataset to diagnose COVID-19. The authors of this study reported their findings in terms of two validation criteria; in the internal validation, the total accuracy was 89%, with 88% precision and 87% sensitivity, whereas in the external validation, it achieved overall accuracy of 79%, with 83% precision and 67% sensitivity. The dataset consisted of 100 pneumonia and healthy images each, whereas only 10 COVID-19 images were used for the validation. Moreover, a paired function pyramid network with an attention module combined with the ResNet-50 approach proposed in [47] and tested using a private dataset consisting of 24 healthy instances and 27 COVID-19 instances achieved 99% AUC and 93% sensitivity. To determine the magnitude of COVID-19, the random forest (RF) technique was suggested in [48], which focused on extensive features extracted using a deep learning algorithm. With 176 images, using three-fold cross-validation, the procedure obtained an average of 87.5% accuracy. A weakly supervised procedure was suggested in [49], where segmentation masks were produced automatically using this technique. For labeling, the CT images and masks were loaded into a 3D CNN. The AUC for this approach was 95.9%.

In summary, most experiments, including those using CXR, rely on inadequate training sets of COVID-19 images from various sources, with no specific protocols. They only re-purpose existing AI-based techniques to solve unique challenges, so AI innovation and clinical utility are minimal. It is difficult to compare research because of the large data variability. Even though all models performed well, it was suggested in [50] that the probability of bias was extreme in all the articles analyzed by the authors, according to the literature in [51]. Algorithms developed for diagnosing COVID-19 using CXR or CT instances perform admirably in general. However, due to data scarcity, some models only use 10 COVID-19 instances in their test set, and only a few models use external validation. As a consequence, they may or may not apply to such contexts. It is important to build a more data-efficient approach to achieve better results on training images. This will permit the incorporation of more images from the unusual class in the test data. The goal of our research is to establish a method that can improve previous models and achieve up-to-date results.

## 3. Methodology

This section introduces the problem statement, dataset and preprocessing procedure used in this study. Then, we present the feature extraction procedure for super resolution. Next, we describe the proposed enhanced Siamese fine-tuned model with a wavelet pooling strategy and modified enhanced super-resolution GAN plus based on low-quality images for COVID-19 identification (COVID-SRSNet). Finally, we provide the implementation details for our proposed model.

### 3.1. Problem Statement

With the continued threat of the pandemic, effective COVID-19 screening is needed. The shortage of COVID-19 test kits and the time taken to produce the samples’ (proper) results in many developed/rural areas pose a significant problem for developing cities with under-equipped hospitals and clinics. Often, developing countries do not have enough COVID-19 kits, limiting primary healthcare clinics’ ability to receive, ship and analyze them, so they must rely on more specialized centers to provide them with the test results. To meet the increasing demand for new test cases, an automated and efficient complementary method is required to respond to the third wave of the pandemic in areas with low access to viral/antibody tests that can be useful in COVID-19.

There are many reports of CT scans being used to find ground-glass opacities and other chest features that are higher in resolution than those of a standard chest X-ray [26]. Thus, because of infection management issues involved with bringing patients to CT units, comparatively high costs (for procurement, installation and repair of CT equipment) and low system availability in developing/rural areas, CT scans cannot be reliable for COVID-19 purposes. On the other hand, a chest X-ray (CXR) may be used to detect COVID-19 [52] and other pneumonia outbreaks, as CXR imaging equipment is readily available in emergency rooms (ERs), public healthcare centers and even remote clinics. However, there are two main bottlenecks in AI-based CXR detection systems for large-scale imaging purposes:

1. Low-resolution (LR) features are a concern.

2. The image inconsistency of the obtained dataset samples also includes blurry and meaningless information. Even for experienced radiologists, analyzing chest X-ray pictures poses challenges in discriminating between the characteristics of COVID-19 pneumonia and community-acquired bacterial pneumonia [52].

Furthermore, the influx of patients into hospital ERs during the pandemic, manual inspection of radiograph data and accurate decision making will contribute to a serious tradeoff regarding accuracy and detection time that can exhaust the radiology unit, and as a result, an automated identification method is needed. COVID-19 third-wave activity would necessitate an increase in portable chest X-ray instruments, as the universal use of these makes CTs redundant. We cover the issues described earlier and present deep learning-based GAN and identification model solutions to solve the third wave problems.

### 3.2. Datasets

There has not yet been any report on the high-quality mining of CXR images for developing COVID-19 diagnosis systems of high clinical value. In this work, we have utilized datasets from two different open sources. The first dataset is taken from the author in [53], which consists of 3616 CXR images of patients diagnosed with COVID-19. In this dataset, we only considered 2980 CXR image cases of COVID-19. The second dataset contains 3029 scans of bacterial pneumonia, 8851 scans of healthy patients and 2983 scans of viral pneumonia obtained from the Kaggle database of RSNA [54]. These collections of different datasets have different variations and dimensions, totaling 11,920 CXR images. Above all, we have only considered 2980 scans for each class for our proposed framework of the classification task on the binary class and multiple classes, respectively, as seen in Table 1. For the binary class, we have considered COVID-19 versus healthy scans, whereas for the multi-class, we have considered four classes, which are COVID-19, healthy, bacterial and viral pneumonia scans. Moreover, since the amount of CXR associated with each class is balanced, the dataset is partitioned into three sets of 40%, 40% and 20% for training, validation and testing, respectively.

#### Image Scale-Based Adaptive Module

**Figure 1:**
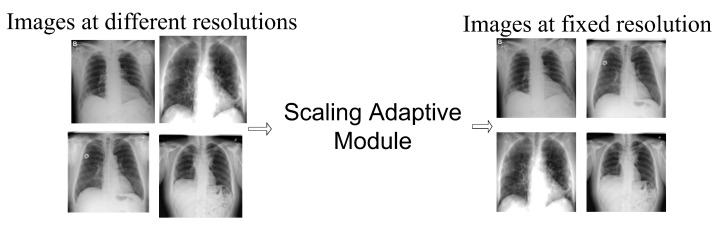
Scaling of images at different resolutions to a fixed resolution using image scaling adaptive module.

Prior to the super-resolution framework, the images were resized using the image scale-based adaptive module. The OpenCV image scaling system was adapted in order to adjust the various resolutions to a fixed dimension. It receives images from different resolutions and converts them to a specific (fixed) resolution of 224×224×3 before passing them to the COVID-SRSNet, as shown in Figure 1.

### 3.3. COVID-SRSNet Identification Architecture

**Figure 2:**
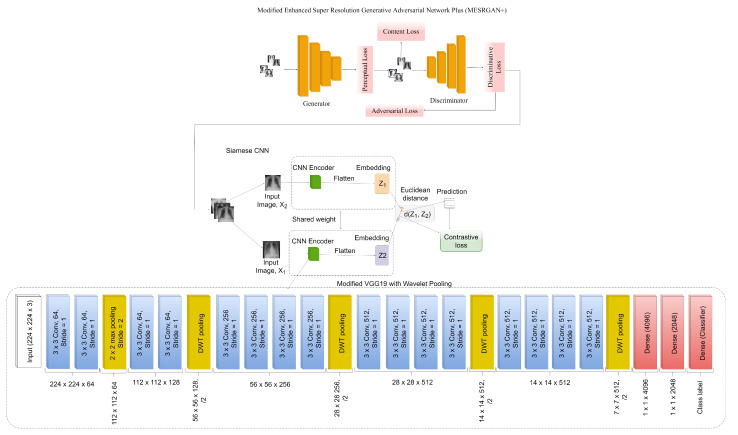
Our proposed modified ESRGAN+ and Siamese convolutional neural network.

The overall illustration of COVID-SRSNet can be seen in Figure 2. It comprises two distinct flows. Before the super-resolution operation, we adopted an image adaptive scaling module as a preprocessing technique to bring the heterogeneous dimensionality of the image resolution to a predefined resolution of 224×224×3, before passing them to the super-resolution network called Modified Enhanced Super Resolution GAN Plus (MESRGAN+). MESRGAN+ is used to convert low-resolution images into high-resolution images and eliminate compression artifacts. Finally, the reconstructed HR images are passed to the Enhanced Siamese Fine-Tuned Model with Wavelet Pooling (ESFMWP) algorithm to obtain and learn discriminative features for the identification of COVID-19. For the sake of simplicity, we refer to the combined frameworks of MESRGAN+ and ESFMWP as COVID-SRSNet.

#### 3.3.1. Modified Enhanced Super-Resolution GAN plus (MESRGAN+)

This study aims at enhancing the overall perception quality of the low-quality chest X-ray images for super resolution before passing them to the proposed Siamese network for COVID-19 identification. In this section, we will present the proposed modified enhanced super-resolution generative adversarial network plus (MESRGAN+) architecture and describe the structural improvement for achieving a balance in perceptual quality and PSNR. Before we discuss our proposed network architecture, we will briefly describe the transition from SRGAN to MESRGAN+.

#### 3.3.2. Transition of Super Resolution by GAN

**Figure 3:**
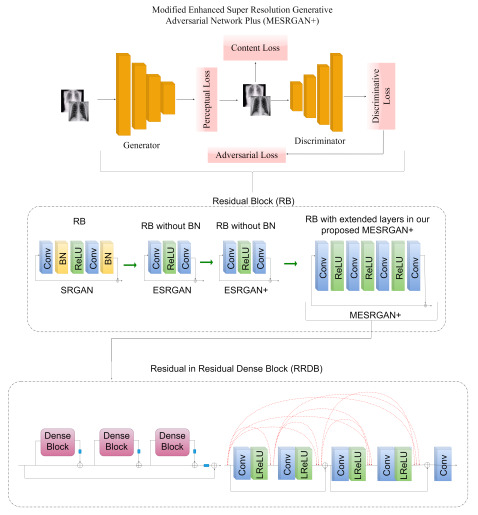
We adopted the fundamental structural configuration of ESRGAN+, where feature extraction and most computation is performed on the LR image feature. We redesigned the structure for better optimization and performance by making a few modifications to the generator structure. The transition from SRGAN to MESRGAN+ is equally showcased.

SRGAN [55] utilizes basic blocks of a deep residual network to recover image-realistic details, in which BN is followed after each convolutional layer, as depicted in Figure 3. The transition from SRGAN to ESRGAN [56] is based on two modifications; the first modification is the removal of all BN in the generator structure and the second modification involves the replacement of the original basic block with a Residual-in-Residual-Dense Block (RRDB), as shown in Figure 3. Finally, the transition from ESRGAN to ESRGAN+ [57] is based on introducing an additional level of residual learning at every two layers inside the dense block, as illustrated in Figure 3, without changing the convolutional structure.

### 3.4. The Proposed MESRGAN+ Architecture

In our proposed super-resolution architecture, the overall structural configuration of the Residual-in-Residual-Dense Block (RRDB) in ESRGAN+ is kept the same, as shown in Figure 3. We made a few modifications to the ESRGAN+ network in the generator structure by extending the convolutional layers. We added two convolutional layers followed by a ReLU activation function. Normally, the direct mapping of the high-dimensional LR features to HR feature vectors ultimately results in high computational complexity, and we know that the dimensions of the LR feature are normally very large. To address this bottleneck, we utilize a 1×1 convolutional layer as the second layer to reduce the computational cost by shrinking the LR dimensional features, thereby maintaining the same kernel size of 64 after the first layer. In order to maintain consistency and the performance of ESRGAN+, we utilized a 3×3 filter size and a kernel size of 64 for the third and fourth convolutional layers.

To produce the high-resolution images from the scale-adaptive module, the scale factor is increased to 4. This image’s network generator produces $v^{k+1}=G_{k}\left(v^{k}\right)$. A feature map is extracted to calculate the perceptual loss before being passed to the final activation function. Pixel-wise loss is measured, and the created image is forwarded to the discriminator network to differentiate between the created image $v^{k+1}$ and the actual image ${\hat{v}}^{k+1}$. This actual image ${\hat{v}}^{k+1}$ is fed to the discriminator network for training, which results in the same super-resolution image $v^{k+1}$. Then, the generator network recalculates the loss function and produces the same image. This entire process was only completed when the discriminator network could no longer distinguish between real and fabricated images. We train the generator function $G_{k}$ to approximate the HR of the next LR image ${\hat{v}}^{k+1}$ that the LR input can represent. In Equation (1), the total super-resolution network is calculated as:

(1) $$\Pi_{Totalloss}=\Pi_{Gen}\left(\Pi_{Perceptualloss}+\mu \Pi_{G}^{Ra}+\eta L1\right)+\Pi_{Dis}^{Ra}$$

As Equation (1) is evaluated, $\Pi_{Gen}$ is the generator loss, $\Pi_{Perceptualloss}$ is the perceptual loss, $\Pi_{G}^{Ra}$ is called the adversarial loss, which is the loss of a relativistic generator, and L1 is the content loss. $\Pi_{Dis}^{Ra}$ is the discriminator loss, and μ and η are coefficients to offset the losses.

Furthermore, noise not only interferes with the reception of images but also affects network quality, so effective noise cancellation is critical to the optimum functionality of image processing pipelines. The JPEG (.jpg) compression format [56] for training the GAN network produces visible objects due to the way in which it decompresses. The solution to this problem is to avoid the use of batch normalization. The method of creating a replacement color for each pixel in the image is based on the concept of using an average similar color of the pixel. It selects a pixel and looks for identical pixels around it, and then computes the Euclidean distance. Finally, the chosen noisy pixel is replaced with the replacement pixel, improving the network’s overall consistency. Removal of unimportant artifacts aids in the extraction of valuable information for Siamese identification.

#### 3.4.1. Perceptual Loss

Perceptual loss works to improve the texture and image accuracy of the generated images [44]. Euclidean distance is used to compare the feature maps of the original image ${\hat{v}}^{k+1}$ and the generated image $v^{k+1}$. According to the definition of [44], the feature map was extracted before using the generator network’s final activation function. In COVID-19 identification, an illumination difference occurs in the CT image datasets obtained from the source. The extraction of feature maps after the activation function causes the model to be inconsistently illuminated, directly impacting the model output. When recapturing HR from LR, it provides close supervision between feature maps. The fact that CT images are not sufficiently HR is well understood, and this aspect boosts model regeneration dramatically. Mapping feature $\alpha_{ij}$ is obtained after *j*th -convolution and before the max-pooling layer. The formality is measured as the distance between the function representations of the super-resolution image $G_{k}\left(v^{k}\right)$ and the real image ${\hat{v}}^{k+1}$. Formal calculation between feature maps is given in algebraic form in Equation (2).

(2) $$\Pi_{Perceptualloss}=\sum_{x=1}^{W_{ij}}\sum_{y=1}^{H_{ij}}{\left(\alpha_{ij}{\left({\hat{v}}^{k+1}\right)}_{xy}-\alpha_{ij}{\left(G_{k}\left(v^{k}\right)\right)}_{xy}\right)}^{2}$$

Instead of penalizing the output image $v^{k+1}$, which is precisely the same as the input image ${\hat{v}}^{k+1}$, perceptual loss prefers the representation to be identical.

#### 3.4.2. Content Loss

By manipulating the HR image $v^{k+1}$ to be close to the ground truth ${\hat{v}}^{k+1}$, the network improves the accuracy at the pixel level by calculating the L1-norm distance between both the ground truth and the recovered image. Compared to the L2 loss, which often results in over-smooth results, the L1 loss is used for better efficiency and convergence. Equation (3) calculates the L1-norm distance between the SR image ${\left(G_{k}\left(v^{k}\right)\right)}_{xy}$ and the ground truth ${\left({\hat{v}}^{k+1}\right)}_{xy}$, given as:

(3) $$Ł1=\sum_{x}^{W}\sum_{y}^{H}{∥\left(G_{k}{\left(v^{k}\right)}_{xy}-{\left({\hat{v}}^{k+1}\right)}_{xy}\right)∥}_{1}$$

#### 3.4.3. Relativistic Loss

The majority of the preliminary research has focused on standard GAN. Meanwhile, we employ a rational discriminative loss in our SR network, ensuring that HR photos are not stylized or unrealistic. In Equation (4), the classification of the images is achieved using the standard discriminator $D_{is}$ in GAN.

(4) $$D_{is}=\sigma (f_{d}\left({\hat{v}}^{k+1}\right))\to 1\;D_{is}=\sigma \left(f_{d}\left(v^{k}\right)\right)\to 0$$

Equation (4) reflects the regular GAN’s operation. $D_{is}$ is the discriminator’s output to classify whether the images are real or artificial. The vector feature discriminator is represented as $f_{d}\left(.\right)$. Additionally, the term “σ” stands for the sigmoid function. Adversarial loss uses a binary classifier to verify whether the obtained result is true or not. We use the relativistic GAN [43] to distinguish between the real ${\hat{v}}^{k+1}$ and created data $G_{k}\left(v^{k}\right)$ with the distance computed as in Equation (5):

(5) $$D_{Ra}({\hat{v}}^{k+1},G_{k}\left(v^{k}\right))$$

RGAN produces images with sharp edges when used in a relativistic model and provides more graphic and detailed information than a typical GAN. It is seen in Equation (6) that RGAN is presented as:

(6) $$\begin{array}{c} D_{Ra}\left(Real,Fake\right)=C\left(Real\right)-E\left(C(Fake)\right)\to 1\\ \mathrm{how}\;\mathrm{realistic}\;\mathrm{an}\;\mathrm{image}\;\mathrm{is}\;\mathrm{compared}\;\mathrm{to}\;a\;\mathrm{fake}\;\mathrm{one}.\\ D_{Ra}\left(Fake,Real\right)=C\left(Fake\right)-E\left(C(Real)\right)\to 0\\ \mathrm{how}\;\mathrm{fake}\;\mathrm{an}\;\mathrm{image}\;\mathrm{is}\;\mathrm{compared}\;\mathrm{to}\;a\;\mathrm{real}\;\mathrm{one}. \end{array}$$

Equation (6) analyzes how realistic an image is compared to an artificial one. Here, E(.) is the average of all real or artificial data in the sample. This slight modification makes the model more efficient than the standard discriminator network. The discriminator network loss is given in Equation (7) defined below:

(7) $$\Pi_{Dis}^{Ra}=-E_{{\hat{v}}^{k+1}}\left[log(D_{Ra}\left({\hat{v}}^{k+1},G_{k}\left(v^{k}\right)\right))\right]-E_{G_{k}\left(v^{k}\right)}\left[log(1-D_{Ra}(G_{k}\left(v^{k}\right),\left({\hat{v}}^{k+1})\right))\right]$$

Despite this, Equation (8) illustrates the adversarial loss for the RGAN.

(8) $$\Pi_{G}^{Ra}=-E_{{\hat{v}}^{k+1}}\left[log(1-D_{Ra}\left({\hat{v}}^{k+1},G_{k}\left(v^{k}\right)\right))\right]-E_{G_{k}\left(v^{k}\right)}\left[log(D_{Ra}(G_{k}\left(v^{k}\right),\left({\hat{v}}^{k+1})\right))\right]$$

The network is concurrently trained for both the actual image ${\hat{v}}^{k+1}$ and created image $G_{k}\left(v^{k}\right)$. To minimize the failure of the discriminator and generator networks, when the discriminator gradient hits its optimum point $(1-D_{{\hat{v}}^{k+1}})\to 0$, i.e., it discriminates between authentic images, it stops learning actual content ${\hat{v}}^{k+1}$ and focuses on generated images $G_{k}\left(v^{k}\right)$. At this level, the custom GAN does not learn how to create more realistic images. In comparison, RGAN studies both images and the gradients are dependent on both terms, i.e., ${\hat{v}}^{k+1}$ and $G_{k}\left(v^{k}\right)$.

### 3.5. Enhanced Siamese Fine-Tuned Model with Wavelet Pooling (ESFMWP)

In the COVID-19 identification network, we propose two similar CNNs with the same weights to learn fixed-length representations. To minimize the computational costs and model complexity, we utilized the fine-tuned VGG19 model by making few modifications to the pooling layers. We only kept the pooling layer of the first block the same, and we replaced every other pooling layer with wavelet pooling in the other blocks, as seen in Figure 2. The fine-tuned modified VGG-19 network is used as the backbone encoder in the Siamese architecture to build feature embeddings from the input images and change the network weights using the pairwise contrastive loss function. We used CXR images from two public datasets to pre-train the embedding CNN network, which generates feature representations that are used by the Siamese network, using metric learning to classify unseen images without retraining. The contributions of our work are summarized as follows:

1. We present framework for diagnosing COVID-19 patients from chest X-ray pictures using COVID-SRSNet.

2. The suggested research examines the advantages of employing a contrastive loss and cross-entropy loss function in the framework’s construction.

3. To improve feature embeddings from the input images, a fine-tuned VGG19 encoder is utilized to capture unbiased feature representations.

4. Performance evaluation is provided to show the usefulness of the proposed framework with a CXR dataset.

To minimize over-fitting, we used 50 percent dropout for regularization. The rectified linear units (ReLU) non-linearity was applied as the activation function, and the learning rate was monitored by the adaptive moment estimation (Adam) optimizer. Euclidean distance was used to evaluate the resemblance between images, and we computed the contrastive loss function, which was then simplified to Equation (9):

(9) $$L\left(W,I_{1},I_{2}\right)=1\;-\;\left(L=0\right)\;*\;\frac{1}{2}D^{2}+1\left(L=1\right)\;*\;\frac{1}{2}\;{\left[max(0,margin-D)\right]}^{2}$$

where $I_{1}$ and $I_{2}$ are similar CNN images. 1(·) is an indicator function that shows whether two images have the same name, where 1 means that they are identical and 0 means that they are different. W represents the mutual parameter vector in neural networks, while $f(I_{1})$ and $f(I_{2})$ represent the latent representation of the input $I_{1}$ and $I_{2}$, respectively. The distance, *D*, between $f(I_{1})$ and $f(I_{2})$ is given in Equation (10).

(10) $$\|f\left(I_{1}\right)\;-\;f\left(I_{2}\right){\|}^{2}$$

Moreover, we examine the binary cross-entropy function as a parameter for comparison with the contrastive loss function. The performance of a classifier with an output probability ranging from 0 to 1 is estimated using binary cross-entropy loss, commonly known as log loss. If the anticipated likelihood differs from the true label, the loss value will rise. This can be expressed as follows in Equation (11), with *y* and *p* being the class label and probability of prediction, respectively:

(11) L=−ylogp+(1−y)log(1−p)

If we supply one training sample from each positive and negative category and aggregate both losses, as shown below in Equation (12), we may train the network to distinguish between similar and dissimilar images.

(12) $$L=L_{pos}+L_{neg}$$

## 4. Experiments

### 4.1. Experimental Setup

To investigate the performance of our proposed model on screening COVID-19, we collected public datasets of chest X-ray images from two open sources. The first dataset is taken from the authors in [53], which consists of 3616 CXR images of patients diagnosed with COVID-19. In this dataset, we only considered 2983 CXR images of COVID-19 cases. The second dataset contains 3029 scans of bacterial pneumonia, 8851 scans of healthy patients and 2983 scans of viral pneumonia obtained from the Kaggle database of RSNA [54]. These collections of different datasets have different variations and dimensions, totaling 11,920 CXR images. Above all, we have only considered 2980 scans for each class for our proposed framework of the classification task on the binary class and multiple classes, respectively. Moreover, we trained the model to differentiate between CXR images of different classes. To execute this task, we made a random selection of N number of images from one class and paired them with another class. This process was repeated until the remaining classes were paired. Each pair contained two images, producing training pairs of 4768 images, validation pairs of 4768 images and test pairs of 2384 images. If the images were the same, we labeled the pair as one; otherwise, we labeled it zero. This is one of the significant advantages of the Siamese neural network. We can generate a large number of training pairs using a relatively smaller number of training images. The L1-norm distance, utilized in this work, calculates the difference between two embeddings. Finally, we used a dense layer with sigmoid activation to predict the output as 0 or 1 depending on whether the two images were similar or not. In our experiment, we conducted a two-class and four-class identification task for verifying the proposed COVID-SRSNet model in the screening task. To verify the effectiveness of our proposed model, we compared our designed COVID-SRSNet model with other up-to-date models.

### 4.2. Implementation Details

In this study, we performed preprocessing to scale the input data to a predefined dimension using the image scaling module. Moreover, we adopted the super-resolution generative adversarial learning technique to address the problem of low quality by generating high-resolution images from the low-resolution counterparts, as well as improving the perceptual quality of the CXR images. The high-resolution imagery was used to construct the COVID-19 identification network. The identification network is a shared weighted Siamese convolutional neural network with the VGG16 pre-trained model as the feature extractor. We fine-tuned the network by replacing the max-pooling layers in each convolutional block with discrete wavelet transform (DWT) pooling, except for the first convolutional block, which retained its max-pooling layer, as illustrated in Figure 2. We added a dropout of 0.5 in the first fully connected (FC) layer to avoid over-fitting. Moreover, another dropout of 0.5 was introduced in the second fully connected layer to avoid over-fitting during the transmission between FC layers. The feature tensor was reduced to 1×1×2048 at the second FC layer. Finally, with the use of the L2-normalization layer, the distance matrix between the feature tensors was computed, followed by a dense layer for classification. Our proposed Siamese model was trained for 30 epochs and a batch size of 16 with the Adam optimizer and a learning rate of 0.002. Moreover, the proposed method has been evaluated in terms of the following metrics: accuracy, precision, sensitivity, specificity, area under the curve and F1 score. The Euclidean distance and contrastive loss function were used to evaluate the resemblance between images and to compute the similarity score. The model was trained on an NVIDIA GTX1080 with Keras as the framework.

### 4.3. Evaluation

The evaluation consists of two parts; first, we illustrate the super-resolution network’s benefits in the image generation process. The second portion of the report involves evaluating the identification network. The evaluation criteria as seen in Equations (13)–(17) was adopted as the metrics to examine the diagnostic performance of our COVID-SRSNet as follows: accuracy (ACC), precision (PRC), sensitivity (SEN), specificity (SPE), F1 score and the area under the curve (AUC).

(13) $$\mathrm{Accuracy}=\frac{\mathrm{TP}+\mathrm{TN}}{\mathrm{TP}+\mathrm{TN}+\mathrm{FP}+\mathrm{FN}}$$

(14) $$\mathrm{Sensitivity}=\frac{\mathrm{TP}}{\mathrm{TP}+\mathrm{FN}}$$

(15) $$\mathrm{Specificity}=\frac{\mathrm{TN}}{\mathrm{TN}+\mathrm{FP}}$$

(16) $$\mathrm{Precision}=\frac{\mathrm{TP}}{\mathrm{TP}+\mathrm{FP}}$$

(17) $$F1-\mathrm{score}=2*\frac{\mathrm{Precision}*\mathrm{Recall}}{\mathrm{Precision}+\mathrm{Recall}}$$

TN, TP, FP and FN represent true negative, true positive, false positive and false negative, respectively.

### 4.4. Super-Resolution Evaluation

**Figure 4:**
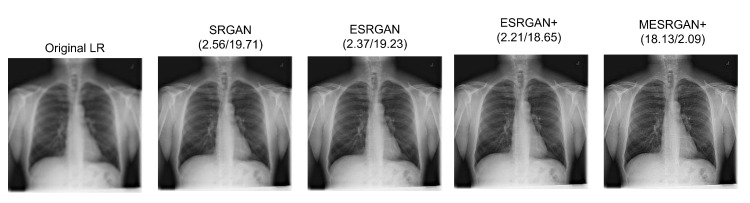
Comparison results of our proposed MESRGAN+ and other selected SOTA models with the same dataset. The PI value is reported on the left and the PSNR is reported on the right.

Here, the aim is to show the efficacy of the super-resolution network for high-resolution imagery tasks. Figure 4 shows the performance of our proposed super-resolution MESRGAN+ and other state-of-the-art models, which are SRGAN, ESRGAN and ERSGAN+. For fair comparison, we employed their available source code with our CXR dataset. One of the aims of this research is to check the PSNR and perceptual index (PI) of the super-resolution models in which our model gives the best results in both cases. MESRGAN+ produces more appropriate images, removes artifacts and improves the extracted features’ clarity by extending the convolutional layer of the generative structure of the residual block and removing batch normalization. Table 2 illustrates the quantitative results of the super-resolution models, and Figure 4 shows the image restoration comparison of our model and other state-of-the-art models.

### 4.5. Loss Function Evaluation

**Figure 5:**
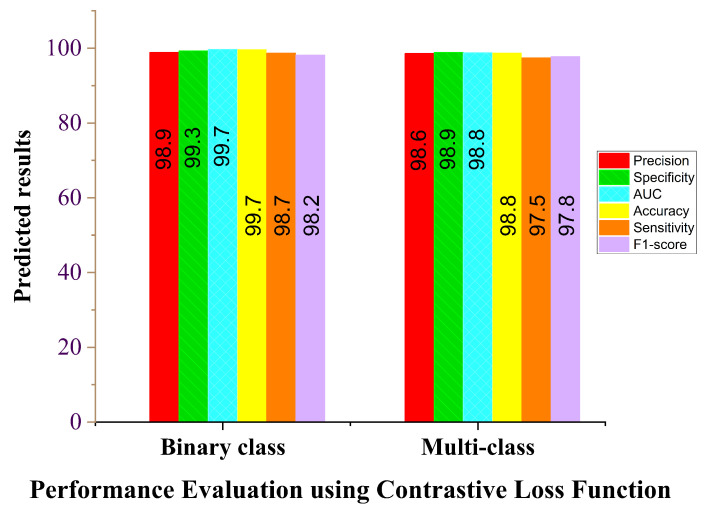
Contrastive loss function report for binary class and multi-class.

**Figure 6:**
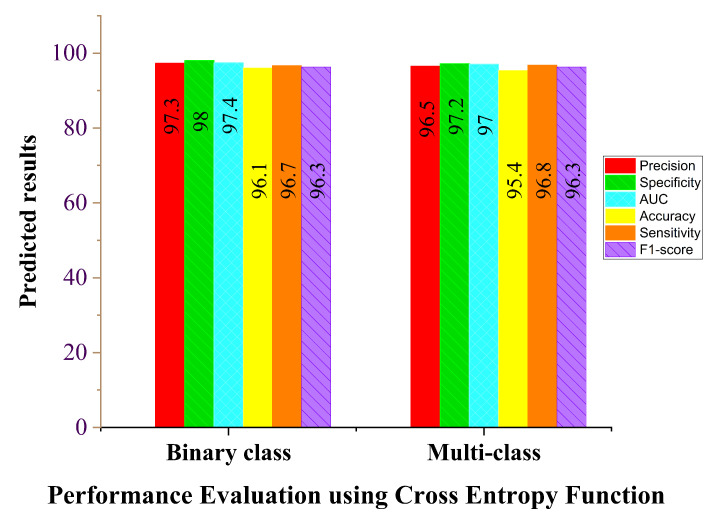
Cross-entropy loss function report for binary class and multi-class.

We evaluated the performance of our proposed COVID-SRSNet model for COVID-19 diagnosis by taking into consideration a few parameter optimizations, such as loss functions. We compared the effect of the cross-entropy loss and contrastive loss function on the overall performance of the model. Since our model is based on the shared weighted technique, we are concerned with how the model performs with different loss functions. To this end, we conducted different experiments to investigate the performance between the cross-entropy and contrastive loss functions in terms of accuracy, AUC, sensitivity, specificity, F1 score and precision. Table 3 illustrates the performance of our proposed model for both binary-class and multiple-class identification problems. The binary class includes COVID-19 and healthy cases, while the multiple class includes COVID-19, healthy, bacterial and viral pneumonia cases. It is evident from the results in and Figure 5 that the model’s accuracy and other performance evaluation metrics increase when using the contrastive loss function for both binary and multiple classes compared to the cross-entropy loss function, as shown in Figure 6. We conducted a similar experiment using the cross-entropy loss function; the results from Figure 6 show that the performance of the model is considerably lower for both binary- and multiple-class identification compared to the contrastive loss function shown in Figure 5, even though it seems to be the common choice for classification tasks. Our proposed model works based on the similarity of image pairs, and the contrastive loss function is presented in this study to be more effective than the cross-entropy loss function.

### 4.6. COVID-19 Identification Evaluation

#### 4.6.1. Result

**Figure 7:**
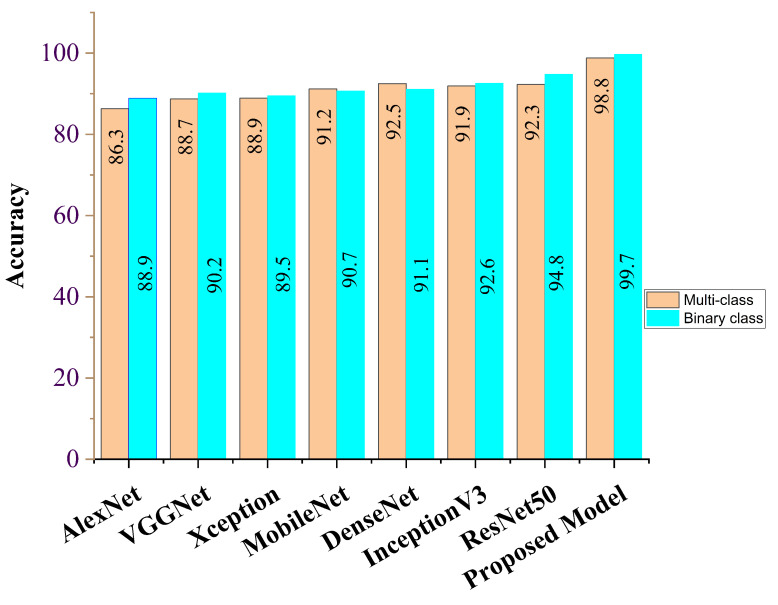
Accuracy report for our proposed model and selected pre-trained models for binary class and multi-class.

**Figure 8:**
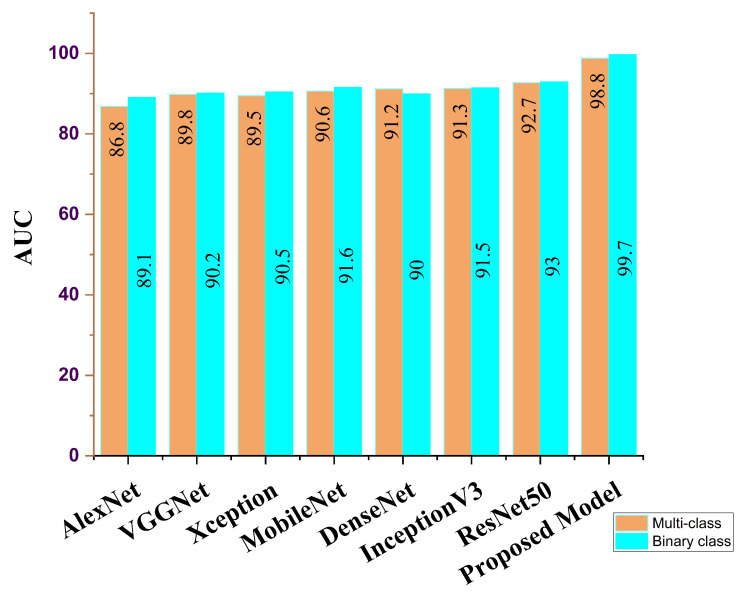
AUC report for our proposed model and selected pre-trained models for binary class and multi-class.

**Figure 9:**
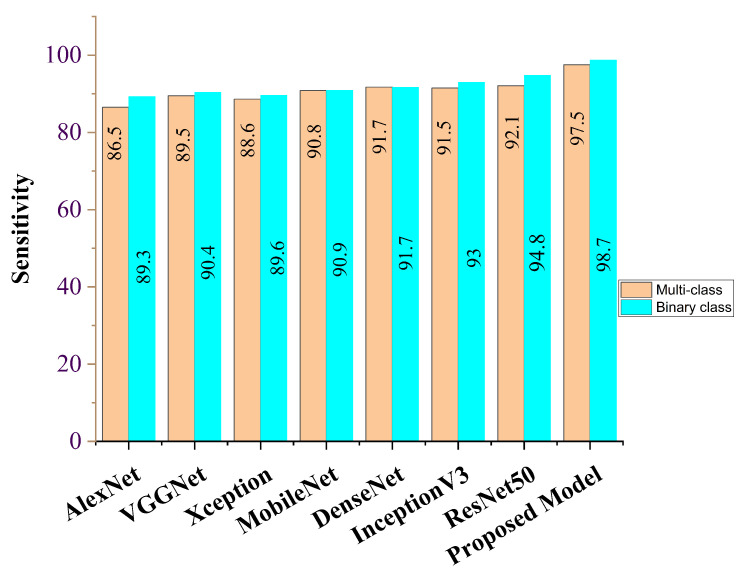
Sensitivity report for our proposed model and selected pre-trained models for binary class and multi-class.

**Figure 10:**
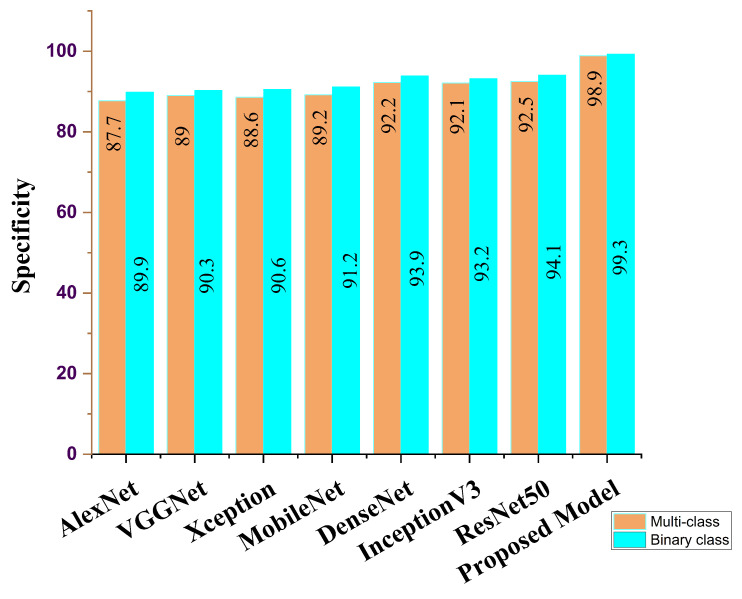
Our proposed MESRGAN+ and Siamese Capsule Network (Siamese-CapsNet).

Our proposed COVID-SRSNet model for COVID-19 identification yields the best results as evaluated on the collected CXR image dataset. Figure 7, Figure 8, Figure 9 and Figure 10 give a clear illustration that our proposed algorithm achieves a promising result in comparison to other state-of-the-art models, including some selected pre-trained models. Our model can help radiologists in the fight against COVID-19, especially with regard to minimizing the low sensitivity result obtained from human-based diagnosis. Our proposed network achieves identification accuracy of 99.7%, precision of 98.9%, sensitivity of 98.7%, specificity of 99.3%, an F1 score of 98.2% and AUC of 99.7% for our binary classification task, as illustrated in Table 4. For multiple classes, our model achieves accuracy of 98.8%, precision of 98.6%, sensitivity of 97.5%, specificity of 98.9%, an F1 score of 97.8% and AUC of 98.8%, as seen in Table 4. Table 5 and Table 6 show the performance comparison of our algorithm with a few selected pre-trained models, including some state-of-the-art COVID-19 models.

**Figure 11:**
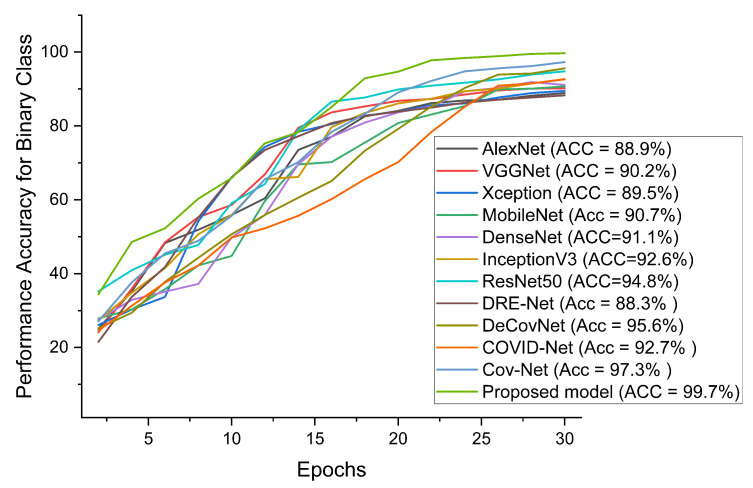
Performance accuracy in comparison with our proposed model and other pre-trained models and selected state-of-the-art COVID-19 models for binary class.

**Figure 12:**
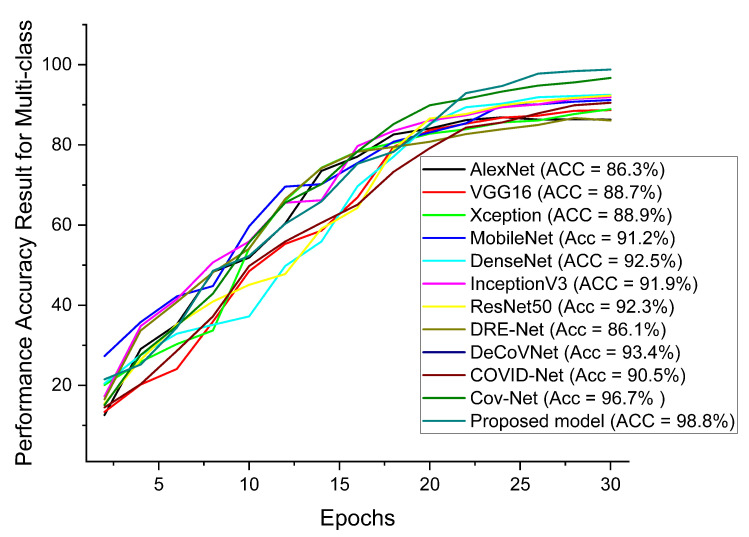
Performance accuracy in comparison with our proposed model and other pre-trained models and selected state-of-the-art COVID-19 models for multi-class.

In addition, our algorithm outperforms all other algorithms based on the performance metrics, as shown in Table 5, Table 6, Table 7, Table 8 and Table 9 for both binary and multiple classification tasks. In as much as there were complex and indistinct lung regions in our CXR images, our algorithm still achieved accurate results, demonstrating its robustness, strength and lower computational cost. Both Figure 11 and Figure 12 illustrate the stability and convergence of the COVID-SRSNet model for COVID-19 diagnosis in both binary and multiple classification tasks. Moreover, the contrastive loss function with the Adam optimizer using a learning rate of 0.002 and epochs of 30 were utilized in the training process.

**Figure 13:**
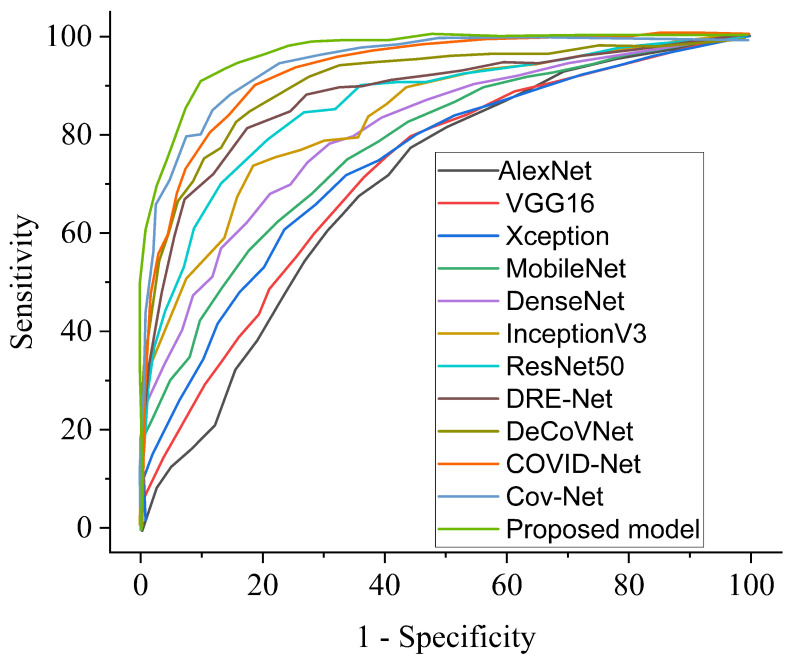
Performance ROC in comparison with our proposed model and other pre-trained models and selected state-of-the-art COVID-19 models for binary class.

**Figure 14:**
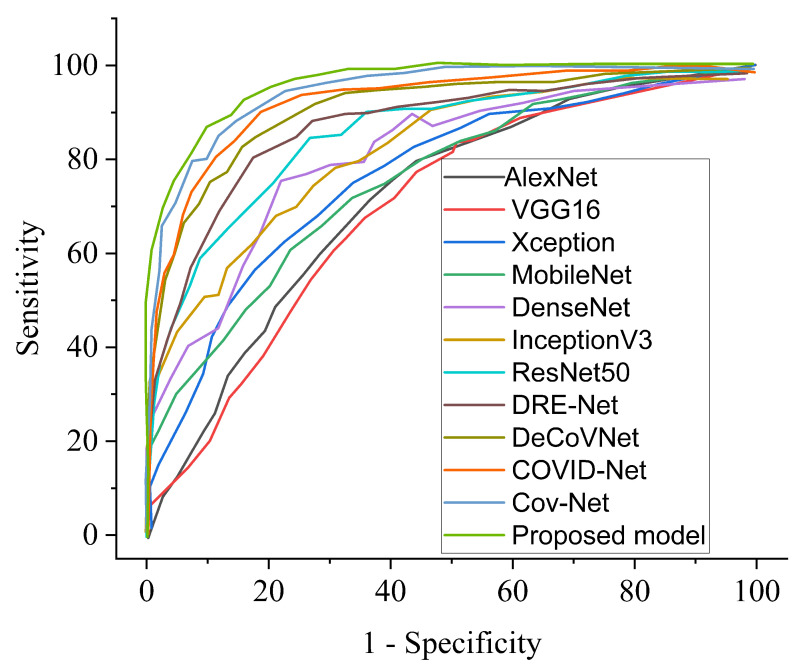
Performance ROC in comparison with our proposed model and other pre-trained models and selected state-of-the-art COVID-19 models for multi-class.

The receiver operating characteristic curve provides a well-informed procedure for decision making. However, our model could offer a better understanding to radiologists in reducing the amount of false positives by balancing the specificity and sensitivity curves, as presented in Figure 13 and Figure 14 for binary and multiple classifications, in comparison with other pre-trained models and a few state-of-the-art COVID-19 models, respectively. The excellent outcome of our proposed model depicts how effective and robust our architecture is, yielding better accuracy in screening COVID-19.

#### 4.6.2. Comparison of Procedures

We compared the findings of our proposed model with those of previous SOTA COVID-19 screening methods, which included Chen et al. [24], Jin et al. [41], Jin et al. [42], Li et al. [28], Shi et al. [40], Song et al. [47], Wang et al. [11], Wang et al. [46], Xu et al. [45] and Zhang et al. [23], on COVID-19 diagnosis tasks, as listed in Table 5. Most research works have laid emphasis on developing new strategies in distinguishing COVID-19 from other forms of pneumonia. Chen et al. [24] constructed a technique to detect COVID-19 based on deep learning from retrospective CT images collected and processed at Renmin Hospital in Wuhan. Their model achieved 95 percent accuracy. Another interesting work proposed by Jin et al. [41] is the timely detection of COVID-19 from CT exams using an AI-based model, with an impressive result of 94 percent accuracy. A medical-based AI algorithm was implemented by Jin et al. [42] to correctly distinguish COVID-19 from other forms of pneumonia using CT exams. This model achieved 92 percent accuracy. Li et al. [28] proposed a COVID-19-based neural network to distinguish COVID-19 from other forms of pneumonia using CT exams, with overall accuracy of 90 percent. Shi et al. [40] proposed an infection region-specific segmentation technique based on a random forest model to distinguish COVID-19 from other forms of pneumonia using CT exams. This study reported 88 percent accuracy. Song et al. [47] proposed a deep learning diagnostic technique based on CT images known as DeepPneumonia, where they utilized 88 CT data of confirmed COVID-19 patients from two hospitals in China. This study reported accuracy of 93 percent. Wang et al. [11] reported a COVID-Net framework tailored to identify COVID-19 from chest radiography. Wang et al. [46] presented a DL framework using CT images for COVID-19 identification. Xu et al. [45] proposed an AI-based technique to screen coronavirus from healthy and viral pneumonia (influenza A) using CT exams. Zhang et al. [23] proposed a method involving a UNet deep learning model based on a weakly supervised technique to correctly examine COVID-19 using 530 CT exams and achieved 90 percent accuracy. An interesting aspect of their study is the adoption of the region of interest as input data. The authors reported a result of 85 percent accuracy.

Nevertheless, the major drawbacks of their papers is that the authors did not consider that real-world CXR images are low-quality in nature and very deep convolutional neural networks may suffer from the vanishing problem, leading to a decline in performance metrics such as accuracy, sensitivity and specificity in COVID-19 screening tasks. To this end, we present a framework that is capable of handling the above challenge and obtains better performance for the diagnosis of COVID-19. Additionally, our proposed COVID-SRSNet model records a high value of 99.7% in accuracy, 99.3% in specificity and 98.7% in sensitivity, which indicates that it is a promising alternative approach to human-based methods of COVID-19 diagnosis since human detection-based methods can lead to a significantly increased rate of false positive outcomes. We presented the receiver operating characteristic curves to assist experts in arriving at a well-informed tradeoff between sensitivity and specificity, which ultimately translates to the relationship between precision and accuracy. Our proposed model is computationally cost-effective, with lower model complexity compared to state-of-the-art models and some famous pre-trained models adopted for comparison, due to the fact that we avoided the use of batch normalization in the residual block of the generator structure in the super-resolution network, while the max-pooling layer in the Siamese convolutional neural network is replaced with wavelet pooling, except for the first convolutional block. We implemented our model using the Keras framework on an NVIDIA GTX 1080. Our model’s complexity is considerably reduced compared to the state-of-the-art models adopted for comparison in this study.

## 5. Discussion

SR imaging holds tremendous promise for practical medical applications. In practice, technical constraints dictated by device components and radiation exposure criteria limit imaging efficiency, necessitating the use of computational methods to improve image resolution. For the identification of COVID-19, our results have demonstrated that the integration of a GAN-based algorithm into the identification network for image super resolution and the proposed Siamese CNN actually improve the overall performance of our proposed model based on low-quality images. Furthermore, using adversarial learning as the SR imaging regularization is a novel mechanism for capturing anatomical details. Current GAN-based methods, on the other hand, introduce additional noise into the produced images. To cope with this limitation, the modified enhanced super-resolution generative adversarial network plus (MESRGAN+) was proposed to learn the complicated deterministic mapping, which improves the quality of images by removing noisy artifacts and inconsistent details. The noise-free SR output from the MESRGAN+ module is fed into the COVID-19 identification Siamese network in an end-to-end framework. Our proposed enhanced Siamese fine-tuned model with wavelet pooling (ESFMWP) network is a similar CNN with the same weights to learn fixed-length representations. The network learns discriminative features and calculates the similarity score to determine whether the pair of input CXR images includes the same scans or not. To achieve a good tradeoff between computational complexity and perceptual quality, we also considered altering a few parameters, such as the filter size, kernel size and the addition of batch normalization in the generator structure of the residual block. It was observed that a smaller filter size and reduced number of kernels help to reduce the model’s complexity and computational cost. However, the perceptual quality did not improve significantly. It is worth mentioning that the addition of batch normalization contributes to the computational complexity of the model and hence reduces the performance of the model in terms of perceptual quality. It also introduces unnecessary distortion, which reduces the generalization capability of the model. Conclusively, training a batch normalization layer under a GAN scheme will most likely introduce artifacts for very deep networks.

Furthermore, a number of researchers have adopted deep convolutional models for identification tasks, but this usually leads to the vanishing gradient problem and huge computational costs. To prevent this problem, we utilized a fine-tuned VGG19 network as the backbone encoder in the Siamese network for feature extraction after making a few modifications, and the L2 regularization term is utilized to regularize the CNN embeddings, while the contrastive loss function and Euclidean distances metric are used to calculate the distances and similarity scores between two CXR scans. It is commonly considered that the decline in performance of image-based COVID-19 diagnosis is because of the low-resolution dataset of CXR. However, this claim is partial because it is possible that deeper convolutional networks perform badly due to exploding vanishing problems. Therefore, we conclude that both the data and architecture are equally responsible for the decline in performance of AI-based COVID-19 diagnosis tasks. To mitigate these issues, we combined a modified enhanced super-resolution generative adversarial network plus (MESRGAN+) without batch normalization in an end-to-end framework with an enhanced Siamese fine-tuned model with wavelet pooling (ESFMWP) network, jointly called COVID-SRSNet, for COVID-19 diagnosis based on low-quality CXR images. In most assessment metrics, the proposed COVID-19 identification network outperforms SOTA methods and pre-trained models, as illustrated in Table 5, Table 6, Table 7, Table 8 and Table 9. Generally, our suggested approach consistently produces better results in terms of accuracy, specificity, sensitivity, precision, F1 score and AUC in both binary and multiple classification tasks.

## 6. Conclusions

In this study, we proposed an enhanced Siamese fine-tuned model with a wavelet pooling strategy and modified enhanced super-resolution GAN plus, jointly called COVID-SRSNet, for a COVID-19 identification framework with the aim of addressing the issue of low quality in COVID-19 CXR images. We implemented an image scaling adaptive module to address the dilemma of resolution variations, while employing our modified enhanced super-resolution generative adversarial network plus (MESRGAN+) to resolve the problem of low-quality CXR images by recapturing high-resolution images from low-resolution counterparts. Then, the generated super-resolution images are passed to the enhanced Siamese fine-tuned model with wavelet pooling (ESFMWP) to learn distinctive features for the COVID-19 identification task. We have demonstrated that our model can create more reasonable and real images, as well as capturing distinct features for COVID-19 identification. By a broad margin, our proposed approach outperforms previous up-to-date COVID-19 diagnostic techniques and some pre-trained models.

This study has some limitations. To begin with, perceptually convincing image reconstruction is a demanding task that will be addressed in the future. The creation of content loss algorithms that characterize image spatial content while being less susceptible to changes in pixel space could improve realistic image SR outcomes even further. Second, the symptoms of COVID-19 may be similar to those of other forms of pneumonia, such as viral pneumonia, bacterial pneumonia and so on. We only compared COVID-19 infection CXR tests to healthy CXR exams for the binary class and other forms of pneumonia for multiple classes,; however, we did not consider CT and ultrasound datasets. For COVID-19 clinical diagnosis, the patient’s contact history, travel history, early symptoms and laboratory assessment are still required. Finally, in as much as our datasets were balanced classes, we will consider imbalanced classes using the GAN-based technique for synthetic images while optimizing our parameters for better performance in our future work. Moreover, we will also consider imbalanced datasets by a synthetic process using GAN-based techniques.

## Figures and Tables

**Table 1:** Distribution of CXR image dataset and their descriptions, showing different categories of pneumonia, healthy and COVID-19 image scans, as well as the number of selected images per category.

Classes	Data Count per Class	Selected No. of Data per Class	Train Set	Val Set	Test Set
Bacteria	3029	2980	1192	1192	596
COVID-19	3616	2980	1192	1192	596
Healthy	8851	2980	1192	1192	596
Virus	2983	2980	1192	1192	596
Total		11,920	4768	4768	2384

**Table 2:** Comparison of the structural configuration of SRGAN, ESRGAN, ESRGAN+ and our proposed MESRGAN+, including their reported peak signal to noise ratio (PSNR) and PI value, using the same CXR dataset.

Parameter	SRGAN	ESRGAN	ESRGAN+	MESRGAN+
Residual block of the generator	Conv(3, 64, 1) Batch norm ReLU Conv(3, 64, 1) Batch norm	Conv(3, 64, 1) ReLU Conv(3, 64, 1)	Conv(3, 64, 1) ReLU Conv(3, 64, 1)	Conv(3, 64, 1) ReLU Conv(1, 64, 1) ReLU Conv(3,64,1) ReLU Conv(3, 64, 1)
Input size	LR	LR	LR	LR
PSNR	19.71 dB	19.23 dB	18.65 dB	18.12 dB
PI	2.56	2.37	2.21	2.09

**Table 3:** Performance evaluation metrics for the proposed model.

Model	ACC (%)	SEN (%)	SPE (%)	AUC (%)	PRE (%)	F1 Score (%)
Cross-entropy binary class	96.1	96.7	98.0	97.4	97.3	96.3
Cross-entropy multi-class	95.4	96.8	97.2	97.0	96.5	96.3
Contrastive loss binary class	99.7	98.7	99.3	99.7	98.9	98.2
Contrastive loss multi-class	98.8	97.5	98.9	98.8	98.6	97.6

**Table 4:** Classification performance of our proposed model based on binary and multiple category tasks.

Performance Metrics	Proposed Model for Binary Class	Proposed Model for Multi-Class
Accuracy (%)	99.7	98.8
Sensitivity (%)	98.7	97.5
Specificity (%)	99.3	98.9
AUC (%)	99.7	98.8
Precision (%)	98.9	98.6
F1 score (%)	98.2	97.8
Time (min)	36.3	39.8

**Table 5:** Comparison of our proposed model with other state-of-the-art COVID-19 screening methods.

SOTA Research Report	Methodology	Performance Evaluation (%)	
Chen et al. [24]	2D UNet ++	ACC	95.2
		SEN	100.0
		SPE	93.6
Jin et al. [41]	2D UNet ++ and 2D CNN	SEN	97.4
		SPE	92.2
Jin et al. [42]	2D CNN	SEN	94.1
		SPE	95.5
Li et al. [28]	2D ResNet-50	SEN	90.0
		SPE	96.0
Shi et al. [40]	Random forest-based CNN	ACC	87.9
		SEN	83.3
		SPE	90.7
Song et al. [47]	2D ResNet-50	SEN	86.0
Wang et al. [11]	2D CNN	ACC	82.9
Wang et al. [46]	3D ResNet and attention	ACC	93.3
		SEN	87.6
		SPE	95.5
Xu et al. [40]	2D CNN	ACC	86.7
Zhang et al. [40]	2D UNet and 2D CNN	SEN	90.7
		SPE	90.7
Ours	MESRGAN+ with Siamese VGGNet for multi-class	ACC	98.8
		SEN	97.5
		SPE	98.9
		AUC	98.8
		PRE	98.6
		F1 score	97.8
Ours	MESRGAN+ with Siamese VGGNet for multi-class	ACC	99.7
		SEN	98.7
		SPE	99.3
		AUC	99.7
		PRE	98.9
		F1 score	98.2

**Table 6:** Comparison of our proposed model with famous pre-trained feature extraction models for multi-class.

Feature Learning Model	ACC (%)	SEN (%)	SPE (%)	AUC (%)	PRE (%)	F1 Score (%)
AlexNet	86.3	86.5	87.7	86.8	86.8	86.9
VGG16	88.7	89.5	88.0	89.8	87.9	89.9
Xception	88.9	88.6	88.6	89.5	88.1	89.8
MobileNet	91.2	90.8	89.2	90.6	89.7	90.5
DenseNet	92.5	91.7	92.2	91.2	91.8	90.8
InceptionV3	91.9	91.5	92.1	91.3	92.1	91.8
ResNet50	92.3	92.1	92.5	92.7	92.5	92.1
Ours	98.8	97.5	98.9	98.8	98.6	97.8

**Table 7:** Comparison of our proposed model with famous pre-trained feature extraction models for binary class using the dataset.

Feature Learning Model	ACC (%)	SEN (%)	SPE (%)	AUC (%)	PRE (%)	F1 Score (%)
AlexNet	88.9	89.3	89.9	89.1	88.2	89.5
VGG16	90.2	90.4	90.3	90.2	89.9	90.1
Xception	89.5	89.6	90.6	90.5	88.6	90.8
MobileNet	90.7	90.9	91.2	91.6	89.8	91.7
DenseNet	91.1	91.7	93.7	90.0	90.8	91.2
InceptionV3	92.6	93.0	93.2	91.5	92.2	92.1
ResNet50	94.8	94.8	94.1	93.0	93.6	93.8
Ours	99.7	98.7	99.3	99.7	98.9	98.2

**Table 8:** Comparison of our proposed model with famous pre-trained feature extraction models for multi-class using the dataset.

COVID-19 Model	ACC (%)	SEN (%)	SPE (%)	AUC (%)	PRE (%)
COVID-Net [11]	90.5	89.2	91.1	89.9	90.0
DRE-Net [47]	86.1	86.7	85.9	86.0	85.8
DeCoVNet [49]	93.4	92.8	93.2	92.1	91.5
Cov-Net [28]	96.7	95.9	96.3	96.1	96.5
Ours	98.8	97.5	98.9	98.8	98.6

**Table 9:** Comparison of our proposed model with some COVID-19 models for binary class using the dataset.

COVID-19 Model	ACC (%)	SEN (%)	SPE (%)	AUC (%)	PRE (%)
COVID-Net [11]	92.7	91.8	92.8	92.2	92.5
DRE-Net [47]	88.3	87.5	88.2	89.4	88.8
DeCoVNet [49]	95.6	95.1	95.8	95.0	95.4
Cov-Net [28]	97.3	97.2	96.9	96.1	97.5
Ours	99.7	98.7	99.3	99.7	98.9

## Data Availability

In this work, we have utilized datasets from two different open sources. The first dataset is taken from the author in [53], which consists of 3616 CXR images of patients diagnosed with COVID-19. In this dataset, we only considered 2980 CXR image cases of COVID-19. The second dataset contains 3029 scans of bacterial pneumonia, 8851 scans of healthy patients and 2983 scans of viral pneumonia obtained from the Kaggle database of RSNA [54].
